# Effect of Imeglimin on mitochondrial function in patients with type 2 diabetes mellitus: a prospective cohort study

**DOI:** 10.3389/fendo.2025.1585834

**Published:** 2025-08-01

**Authors:** Abhishek Satheesan, Janardanan Kumar, Kakithakara Vajravelu Leela, Ria Murugesan

**Affiliations:** ^1^ Department of Microbiology, SRM Medical College Hospital and Research Centre, SRM Institute of Science and Technology (SRMIST), Kattankulathur, Chengalpattu, Tamil Nadu, India; ^2^ Department of General Medicine, SRM Medical College Hospital and Research Centre, SRM Institute of Science and Technology (SRMIST), Kattankulathur, Chengalpattu, Tamil Nadu, India

**Keywords:** Imeglimin, mitochondrial DNA, telomere length, type 2 diabetes, glycemic control

## Abstract

**Background:**

Imeglimin, a novel oral hypoglycemic agent, is known to influence mitochondrial function and glucose metabolism. This study evaluates its effects on glycemic control, mitochondrial DNA (mtDNA) copy number, and telomere dynamics in type 2 diabetes mellitus (T2DM).

**Methods:**

Type 2 diabetes mellitus patients were assigned to one of four treatment groups: (1) Imeglimin alone, (2) Imeglimin with metformin, (3) Imeglimin with other oral hypoglycemic agents, and (4) Metformin with other oral hypoglycemic agents. Clinical and metabolic parameters, mtDNA copy number, and relative telomere length were assessed at baseline and six months. Statistical analyses included paired t-tests and mixed models.

**Results:**

The study included participants with a mean age of 55.6 years (57% male, BMI 28.8 kg/m^2^). HbA1c significantly decreased in the Imeglimin + Other OHA (p < 0.001), Imeglimin + Metformin (p < 0.001), and Metformin + Other OHA (p < 0.001) groups, with a smaller but significant decrease in the Imeglimin monotherapy group (p = 0.04). mtDNA copy number increased significantly in the Imeglimin-based combination groups (p < 0.05) but not with monotherapy (p = 0.18). No serious adverse events were reported. Relative telomere length was only associated with age and changes in LDL-c levels.

**Conclusion:**

Imeglimin-based combination therapy effectively improves glycemic control and mitochondrial function, while monotherapy offers limited benefits. Combination therapy may be preferable for optimizing metabolic outcomes in T2DM. No significant change in telomere length was observed during the short period of time.

## Introduction

Type 2 diabetes mellitus (T2DM) is characterized by chronic hyperglycemia, insulin resistance, and progressive β-cell dysfunction, leading to systemic metabolic complications (1, 2). Mitochondrial dysfunction plays a crucial role in the pathogenesis of T2DM, contributing to impaired glucose metabolism, oxidative stress, and cellular senescence (3). Emerging evidence suggests that mitochondrial DNA (mtDNA) copy number, a marker of mitochondrial function, is reduced in individuals with diabetes, reflecting compromised mitochondrial biogenesis and increased oxidative damage (4, 5). Additionally, telomere attrition, a hallmark of cellular aging, has been associated with T2DM, insulin resistance, and metabolic decline, indicating a potential interplay between mitochondrial health and telomere dynamics in diabetes progression (6–8).

Imeglimin, a novel oxidative phosphorylation modulator, has demonstrated beneficial effects in improving mitochondrial function and enhancing β-cell survival. Unlike traditional antidiabetic agents, Imeglimin exerts its effects by targeting mitochondrial bioenergetics, reducing reactive oxygen species (ROS) production, and preserving cellular integrity (9, 10). While preclinical studies have shown that Imeglimin improves mitochondrial efficiency and β-cell function, its impact on mtDNA copy number and telomere dynamics in T2DM patients remains unexplored (11–13). Given the crucial role of mitochondrial health in cellular aging and diabetes progression, investigating whether Imeglimin therapy can ameliorate mitochondrial dysfunction and telomere attrition is essential.

This longitudinal prospective study aims to evaluate the effects of Imeglimin therapy on mtDNA copy number and telomere length in individuals with T2DM. By assessing these parameters over time, we seek to determine whether Imeglimin can mitigate mitochondrial dysfunction and delay telomere shortening, thereby providing novel insights into its potential as a disease-modifying therapy in diabetes management. Understanding the influence of Imeglimin on these fundamental cellular processes may offer new therapeutic strategies for preserving metabolic health and delaying diabetes-related complications.

## Methods

### Study design and population

This longitudinal prospective cohort study was conducted at SRM Medical College Hospital and Research Centre, Kattankulathur, approved by the institutional ethics committee (IEC No: 8708/IEC/2023), Informed consent was obtained from all participants. The study was registered under Clinical Trial Registry of India on 27/12/2023 (CTRI/2023/12/060844).

### Pharmacological intervention

Imeglimin was administered orally at a dose of 1,000 mg twice daily (total daily dose 2,000 mg), consistent with recommended dosing guidelines. Metformin was administered at standard doses ranging from 500 mg to 2,000 mg per day, given in one or two divided doses, depending on individual glycemic control and tolerance. All treatments were administered at standard clinical doses following local guidelines. Participants in the Imeglimin monotherapy group received this as their initial anti-diabetic treatment, while those in combination therapy groups received Imeglimin added to their existing baseline medications. Medication adherence was monitored throughout the study duration. Participants in the Imeglimin Monotherapy group were drug-naïve patients with no prior anti-hyperglycemic treatment. In contrast, patients in the Imeglimin + Metformin and Imeglimin + other OHA groups had uncontrolled HbA1c despite baseline therapy, so Imeglimin was added to their existing treatment regimens. The Metformin + other OHA group consisted of patients continuing Metformin alongside other oral hypoglycemic agents, which included sulfonylureas, DPP-4 inhibitors and SGLT2 inhibitors. All treatments were administered at standard clinical doses following local guidelines.

#### Inclusion criteria

- Adults aged 18–70 years.
- Diagnosed T2DM with HbA1c levels of 7.0–10.0%.
- Stable medication regimen for at least three months.

#### Exclusion criteria

- Insulin therapy.
- Advanced diabetic complications.
- Pregnancy or lactation.
- Chronic diseases affecting mtDNA or telomere dynamics.

#### Study groups

Participants were stratified based on prescribed therapies:

- Imeglimin monotherapy (Group 1).
- Imeglimin + metformin (Group 2).
- Imeglimin + other OHAs (Group 3).
- Metformin + other OHAs (Control, Group 4).

#### Follow-up schedule

**Figure 1:**
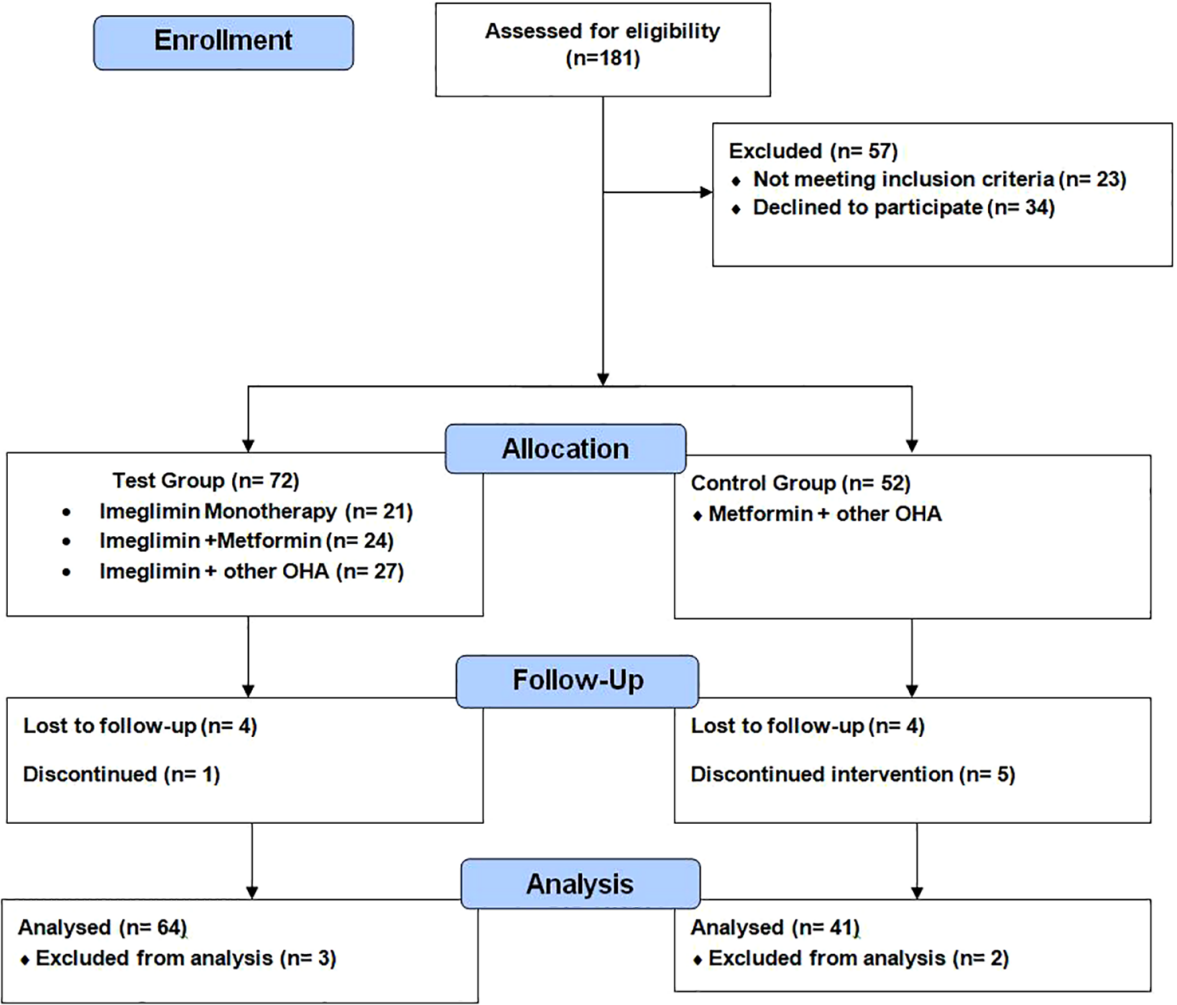
CONSORT flow diagram: enrollment, allocation, follow-up, and analysis of participants in a prospective observational study evaluating Imeglimin in type 2 diabetes mellitus.

Participants were assessed at baseline, and after 6 months (**Figure 1**).

### Data collection

#### Clinical parameters

- HbA1c: Measured using high-performance liquid chromatography (HPLC).
- Lipid profile (LDL, HDL, triglycerides): Assessed using enzymatic colorimetric assays.
- BMI: Calculated as weight in kilograms divided by the square of height in meters.

#### Biomarker assessment

• mtDNA copy number:

Quantified using qPCR with specific primers targeting mitochondrial and nuclear genes.

To determine mitochondrial DNA copy number (mtDNA-CN), whole blood samples are collected in EDTA tubes, and genomic DNA is extracted using a standardized extraction kit. Quantitative PCR (qPCR) is employed to amplify mitochondrial DNA (e.g., D-loop region) and nuclear DNA (e.g., β2-microglobulin, B2M) as a reference.

The primers used for amplification are:

mt-ND1 (targeting the mitochondrial ND1 gene):

Forward: GAGCGATGGTGAGAGCTAAGGT
Reverse: CCCTAAAACCCGCCACATCT

B2M (targeting the nuclear beta-2-microglobulin gene):

Forward: CCAGCAGAGAATGGAAAGTCAA
Reverse: TCTCTCTCCATTCTTCAGTAAGTCAACT

The mt-ND1 primers are specific to the mitochondrial ND1 gene, enabling the quantification of mtDNA, while the B2M primers are designed to amplify the B2M gene as a reference for nuclear DNA. These primers enable the relative quantification of mtDNA to nuclear DNA in qPCR assays. The qPCR protocol begins with an initial denaturation at 95°C for 5 minutes, followed by 40 cycles of denaturation at 95°C for 15 seconds, annealing at 60°C for 30 seconds, and extension at 72°C for 30 seconds. The procedure concludes with a final extension at 72°C for 5 minutes (14).

• Relative telomere length:

Peripheral blood samples (2 mL) were obtained from participants following an overnight fast. Relative telomere length (RTL) in leukocytes was analyzed using quantitative PCR (qPCR), which calculates the telomere repeat copy number relative to the single-copy gene copy number (T/S ratio). The primer sequences used were:

Forward (Telomere): 5’- GGTTTTTGAGGGTGAGGGTGAGGGTGAGGGTGAGGGT-3’
Reverse (Telomere): 5’-TCCCGACTATCCCTATCCCTATCCCTATCCCTATCCCTA-3’

For 36B4, primers were:

Forward (36B4): 5’-CAGCAAGTGGGAAGGTGTAATCC-3’
Reverse (36B4): 5’-CCCATTCTATCATCAACGGGTACAA-3’

• The protocol adhered to Cawthon’s method, using qPCR to compare the amplification of telomere sequences to that of the 36B4 gene. Each 20 µL reaction mixture contained 7–10 ng of DNA, SYBR Green dye, and specific primers targeting both telomeres and 36B4. The PCR conditions included an initial denaturation at 95°C for 10 minutes, followed by 25–30 cycles of 95°C for 15 seconds and 54°C for 2 minutes for telomere amplification. For 36B4 amplification, 30–35 cycles were performed at 95°C for 15 seconds and 58–60°C for 1 minute. The T/S ratio was calculated using the 2−ΔΔCt method (15).

### Ethical considerations

This research was carried out in accordance with ethical standards and received approval from the Institutional Ethics Committee of SRM Medical College Hospital and Research Centre, SRMIST, Kattankulathur. Prior to participation, informed consent was obtained from all individuals, guaranteeing voluntary involvement and data confidentiality. All procedures complied with the ethical principles set forth in the Declaration of Helsinki for research involving human subjects.

### Statistical analysis

Statistical analysis was performed using SPSS version 25. Normality of data was assessed using the Shapiro-Wilk test. Paired t-tests or Wilcoxon signed-rank tests were used to evaluate within-group changes over 6 months, while one-way ANOVA or Kruskal-Wallis tests were applied for between-group comparisons, depending on data distribution. *Post hoc* analyses were conducted with Bonferroni or Dunn’s correction as appropriate. To assess changes in HbA1c over time and across treatment groups, a linear mixed model was used, including fixed effects for time, treatment group, and their interaction. A p-value of <0.05 was considered statistically significant.

## Results

According to **Table 1**, the average participant age was 55.6 ± 6.9 years, with 57% being male. The mean BMI was 28.8 ± 3.4 kg/m^2^, indicating an overweight population. Baseline HbA1c averaged 8.0 ± 0.88%, with significantly lower values in the Imeglimin + Metformin (7.5 ± 0.6%) and Imeglimin + Other OHAs (7.4 ± 0.3%) groups compared to the Imeglimin monotherapy and control groups (p = 0.001), suggesting better glycemic control in the combination therapy arms. No significant differences were observed between groups for total cholesterol, LDL-C, HDL-C, and triglycerides (reported as median with interquartile range). Relative telomere length and mtDNA copy number (also expressed as medians with IQR) were comparable across groups (p = 0.39 and 0.45, respectively), supporting baseline equivalence. No serious adverse events occurred; only two participants (1.1%) reported mild shoulder pain, which did not require intervention or discontinuation.

**Table 1:** Baseline demographic and clinical characteristics of study participants.

Variable	Imeglimin monotherapy (n=19)	Imeglimin + metformin (n=21)	Imeglimin + other OHAs (n=24)	Metformin + other OHA (n=41)	p-value
Age (years, mean ± SD)	55.2 ± 6.5	56.8 ± 7.2	54.9 ± 6.8	55.7 ± 7.0	0.68
Sex (Male, %)	58	54	56	60	0.88
BMI (kg/m^2^, mean ± SD)	28.5 ± 3.2	29.0 ± 3.5	28.7 ± 2.4	28.9 ± 5.6	0.79
Total Cholesterol (mg/dL, mean ± SD)	180.5 ± 30.2	167.8 ± 31.4	190.1 ± 21.3	184.6 ± 19.3	0.71
LDL-C (mg/dL, mean ± SD)	110.2 ± 25.1	112.5 ± 27.3	111.8 ± 26.5	113.1 ± 24.8	0.83
VLDL-C (mg/dL, mean ± SD)	26.5 ± 6.1	27.1 ± 5.8	26.9 ± 5.1	27.0 ± 6.2	0.90
HDL-C (mg/dL, mean ± SD)	42.8 ± 7.2	43.5 ± 6.8	42.9 ± 7.0	41.8 ± 6.9	0.77
Triglycerides (mg/dL, median [IQR])	135 (120–150)	140 (125–155)	138 (122–152)	136 (123–150)	0.62
HbA1c (%, mean ± SD)	7.6 ± 1.1	7.9 ± 0.6	8.1 ± 0.3	8.5 ± 1.2	0.001
Serum Creatinine (mg/dL, mean ± SD)	0.92 ± 0.15	0.94 ± 0.16	0.91 ± 0.14	0.93 ± 0.15	0.68
UACR (mg/g, median [IQR])	35 (25–50)	38 (27–55)	36 (28–53)	37 (26–54)	0.59
Relative Telomere Length (median [IQR])	0.85 (0.78–0.92)	0.83 (0.76–0.89)	0.84 (0.77–0.90)	0.81 (0.74–0.88)	0.39
mtDNA Copy Number (mean ± SD)	36.95 ± 4.31	38.42 ± 4.94	37.7 ± 5.51	36.12 ± 4.79	0.45

Continuous variables are presented as mean ± standard deviation (SD) for normally distributed data or as median [interquartile range (IQR)] for non-normally distributed data. BMI, Body Mass Index; LDL-C, Low-Density Lipoprotein Cholesterol; VLDL-C, Very-Low-Density Lipoprotein Cholesterol; HDL-C, High-Density Lipoprotein Cholesterol; HbA1c, Glycated Hemoglobin; UACR, Urinary Albumin-to-Creatinine Ratio; mtDNA, Mitochondrial DNA. Statistical tests used include one-way ANOVA for normally distributed continuous variables, the Kruskal-Wallis test for non-normally distributed continuous variables, and the chi-square test for categorical variables. Normality was assessed using the Shapiro-Wilk test.

Both mtDNA copy number and relative telomere length (RTL) showed significant inverse correlations with age, indicating a decline in mitochondrial and telomere integrity with aging. A negative correlation was found between mtDNA copy number and HbA1c reduction (r = -0.30, p = 0.006), suggesting improved glycemic control is linked to smaller mtDNA changes. RTL also inversely correlated with LDL-C changes (r = -0.24, p = 0.027), implying a potential relationship between telomere dynamics and lipid metabolism. Other metabolic parameters, including BMI, UACR, and serum creatinine, did not show any significant correlations with mitochondrial or telomere markers (**Table 2**).

**Table 2:** Correlation analysis between changes in mitochondrial and telomere parameters with metabolic markers.

Parameter	Δ mtDNA copy number		Δ RTL	
	r	p-value	r	p-value
Age	-0.26	0.017*	-0.22	0.039*
Gender	+0.08	0.342	+0.06	0.414
Diabetes Duration	-0.15	0.112	-0.10	0.238
Δ HbA1c	-0.30	0.006*	-0.11	0.189
Δ Total Cholesterol	-0.13	0.157	-0.09	0.276
Δ LDL-C	-0.12	0.192	-0.24	0.027*
Δ HDL-C	+0.10	0.221	+0.08	0.312
Δ Triglycerides	-0.28	0.009*	-0.14	0.129
Δ BMI	-0.16	0.097	-0.07	0.343
Δ UACR	-0.18	0.073	-0.13	0.144
Δ Serum Creatinine	-0.12	0.188	-0.10	0.254

Δ, Change in parameter; r, Pearson correlation coefficient.; p, p-value indicating statistical significance.; *p < 0.05 indicates statistical significance.

Subgroup analysis based on baseline HbA1c (<7.5% vs. ≥7.5%) revealed that patients with HbA1c <7.2% exhibited greater increases in mtDNA copy number than those with HbA1c ≥7.2%. The most significant increases were observed in the Imeglimin + OHA group (p = 0.029), followed by Imeglimin + Metformin (p = 0.035) and Imeglimin monotherapy (p = 0.041). Patients with baseline HbA1c ≥7.2% showed smaller mtDNA increases across all treatment groups. These findings suggest lower baseline HbA1c (<7.2%) is associated with greater mtDNA copy number increases in patients receiving Imeglimin-based therapies.

Paired t-test analysis assessed within-group changes in metabolic parameters over six months across the four treatment arms (**Table 3**). HbA1c significantly decreased in all groups: modestly in the Imeglimin monotherapy group (p < 0.05) and more markedly in the combination therapy groups (p < 0.001) compared to baseline values, indicating greater glycemic improvements with combination treatments.

**Table 3:** Paired t-test analysis of metabolic parameters over 6 months.

Parameter	Group	Mean Difference (Δ)	t- value	df	p-value	95% CI for Δ
HbA1c (%)	Imeglimin monotherapy	-0.3 ± 0.8	2.1	48	0.04*	(0.02, 0.58)
	Imeglimin + Metformin	-0.8 ± 0.6	-5.4	48	<0.001**	(-1.10, -0.50)
	Imeglimin + other OHA	-0.9 ± 0.7	-6.0	48	<0.001**	(-1.25, -0.55)
	Metformin + other OHA	-0.5 ± 0.5	-4.8	48	<0.001**	(-0.95, -0.40)
BMI (kg/m²)	Imeglimin monotherapy	-0.2 ± 0.5	-1.5	48	0.13	(-0.50, 0.10)
	Imeglimin + Metformin	-0.5 ± 0.7	-2.8	48	0.07	(-0.90, -0.10)
	Imeglimin + other OHA	-0.6 ± 0.6	-3.2	48	0.05*	(-0.95, -0.20)
	Metformin + other OHA	-0.4 ± 0.8	-2.1	48	0.08	(-0.85, - 0.05)
Triglycerides (mg/dL)	Imeglimin monotherapy	-10.2 ± 20.5	-1.8	48	0.09	(-20.5, 1.5)
	Imeglimin + Metformin	-18.5 ± 22.3	-2.5	48	0.06	(-30.2, -5.2)
	Imeglimin + other OHA	-15.8 ± 18.9	-2.0	48	0.07	(-25.5, -3.0)
	Metformin + other OHA	-12.3 ± 19.5	-1.9	48	0.08	(-23.0, -2.0)
Serum Creatinine (mg/dL)	Imeglimin monotherapy	+0.02 ± 0.05	1.3	48	0.19	(-0.01, 0.05)
	Imeglimin + Metformin	-0.01 ± 0.04	-1.0	48	0.26	(-0.03, 0.01)
	Imeglimin + other OHA	-0.02 ± 0.03	-1.5	48	0.17	(-0.04, 0.00)
	Metformin + other OHA	-0.01 ± 0.03	-1.2	48	0.22	(-0.03, 0.01)
UACR (mg/g)	Imeglimin monotherapy	-1.8 ± 8.5	-0.9	48	0.34	(-5.0, 1.2)
	Imeglimin + Metformin	-4.5 ± 9.2	-1.8	48	0.11	(-8.5, -0.5)
	Imeglimin + other OHA	-3.7 ± 7.8	-1.6	48	0.14	(-7.5, 0.1)
	Metformin + other OHA	-2.9 ± 6.9	-1.4	48	0.18	(-6.5, 0.2)
mtDNA Copy Number	Imeglimin monotherapy	-1.5 ± 2.8	-2.2	48	0.18	(-2.9, -0.1)
	Imeglimin + Metformin	+2.4 ± 3.1	3.1	48	0.004*	(0.8, 4.0)
	Imeglimin + other OHA	+2.2 ± 2.9	2.9	48	0.005*	(0.7, 3.8)
	Metformin + other OHA	+1.9 ± 2.7	2.5	48	0.01*	(0.5, 3.3)
Relative Telomere Length	Imeglimin monotherapy	-0.02 ± 0.05	-1.0	48	0.32	(-0.05, 0.01)
	Imeglimin + Metformin	-0.01 ± 0.04	-0.8	48	0.42	(-0.04, 0.01)
	Imeglimin + other OHA	-0.02 ± 0.05	-1.2	48	0.28	(-0.05, 0.01)
	Metformin + other OHA	-0.01 ± 0.04	-0.9	48	0.36	(-0.04, 0.01)

Δ, Mean difference; t, t-statistic from paired t-test; df, degrees of freedom; CI, confidence interval; *p < 0.05, **p < 0.001 indicate statistical significance.

MtDNA copy number increased significantly in all combination groups (p < 0.05) compared to baseline values but not in the monotherapy group (p = 0.18), suggesting a differential impact of combination therapy on mitochondrial health. No significant within-group changes were observed for BMI, triglycerides, serum creatinine, UACR, or relative telomere length (p > 0.05). These trends are further examined in between-group comparisons using mixed-model analysis in the following section.

The linear mixed model showed that baseline mtDNA copy number levels did not differ significantly between treatment groups, indicating comparable starting points. Over six months, significant within-group increases in mtDNA copy number were observed in the Imeglimin + Other OHA (β = +1.30, p < 0.001), Imeglimin + Metformin (β = +1.00, p < 0.001), and Metformin + Other OHA (reference group; β = +0.90, p = 0.048) groups, demonstrating improved mitochondrial function with these regimens. The Imeglimin monotherapy group showed a smaller, non-significant increase (β = +0.40, p = 0.112), suggesting less pronounced mitochondrial benefit from monotherapy.

*Post hoc* analysis at 6 months revealed that both Imeglimin + Other OHA (+0.40, p = 0.009) and Imeglimin + Metformin (+0.30, p = 0.035) groups had significantly higher mtDNA copy numbers compared to the reference (Metformin + Other OHA). The Imeglimin Monotherapy group showed no significant difference (+0.10, p = 0.44). Also, Imeglimin + Other OHA had significantly higher mtDNA than Imeglimin monotherapy (+0.30, p = 0.035). These findings indicate superior mitochondrial improvement with Imeglimin-based combinations. (**Table 4**).

**Table 4:** Linear mixed model results and *post hoc* pairwise comparisons of mtDNA copy number at 6 months.

Parameter/Comparison	Estimate	Std. error	t-value	p-value
LMM Fixed Effects				
Intercept	37.85	2.73	13.87	<0.001**
Time (Baseline vs. 6 months)	0.35	0.18	1.94	0.054
Group: Imeglimin Monotherapy	-0.20	1.10	-0.18	0.856
Group: Imeglimin + Other OHA	1.10	1.10	1.00	0.319
Group: Imeglimin + Metformin	0.75	1.10	0.68	0.497
Time × Imeglimin Monotherapy	0.40	0.25	1.60	0.112
Time × Imeglimin + Other OHA	1.30	0.25	5.20	<0.001**
Time × Imeglimin + Metformin	1.00	0.25	4.00	<0.001**
Time × Metformin + Other OHA (Reference)	0.90	1.28	0.70	0.048*
*Post Hoc* Pairwise Comparisons at 6 months				
Imeglimin + Other OHA vs. Reference	+0.40	0.15	2.67	0.009*
Imeglimin + Metformin vs. Reference	+0.30	0.14	2.14	0.035 *
Imeglimin Monotherapy vs. Reference	+0.10	0.13	0.77	0.44
Imeglimin + Other OHA vs. Imeglimin Monotherapy	+0.30	0.14	2.14	0.035 *
Imeglimin + Metformin vs. Imeglimin Monotherapy	+0.20	0.14	1.43	0.16

Estimate: Coefficient representing the effect size in the linear mixed model, Std. Error, Standard error of the estimate, Reference = Metformin + other OHA group, t: t-statistic from the linear mixed model, *p < 0.05, **p < 0.001 indicate statistical significance.

Together, these findings indicate that while all combination therapies improved mitochondrial function over time, Imeglimin-based combination treatments produced superior mitochondrial benefits both within groups over time and between groups at 6 months, compared to standard regimens or Imeglimin monotherapy.

The linear mixed model indicated a significant overall reduction in HbA1c levels across all groups over 6 months (β = -0.40, *p* < 0.001). Baseline HbA1c values were similar across groups, with no significant differences at baseline. Among treatment groups, Imeglimin + Other OHA showed a significant main group effect at baseline (β = -0.25, *p* = 0.039), while the other groups did not differ significantly from the reference. Interaction terms revealed that the Imeglimin + Other OHA (β = -0.72, *p* < 0.001) and Imeglimin + Metformin (β = -0.64, *p* < 0.001) groups experienced significantly greater HbA1c reductions over time compared to the reference group, indicating stronger within-group improvements. The Imeglimin monotherapy group showed a smaller, non-significant HbA1c decline over time (β = -0.15, *p* = 0.136) (**Table 5**).

**Table 5:** Linear mixed model results and *post hoc* pairwise comparisons of HbA1c levels at 6 months.

Parameter/Comparison	Estimate	Std. error	t-value	p-value
LMM Fixed Effects				
Intercept	8.20	0.15	54.67	<0.001**
Time (Baseline vs. 6 months)	-0.40	0.08	-5.00	<0.001**
Group: Imeglimin Monotherapy	-0.05	0.12	-0.42	0.678
Group: Imeglimin + Other OHA	-0.25	0.12	-2.08	0.039*
Group: Imeglimin + Metformin	-0.20	0.12	-1.67	0.098
Time × Imeglimin Monotherapy	-0.15	0.10	-1.50	0.136
Time × Imeglimin + Other OHA	-0.72	0.10	-7.00	<0.001**
Time × Imeglimin + Metformin	-0.64	0.10	-6.00	<0.001**
Time × Metformin + Other OHA (Reference)	-0.69	0.10	-3.00	0.003*
*Post Hoc* Pairwise Comparisons at 6 months				
Imeglimin + Other OHA vs. Reference	-0.28	0.09	-3.11	0.004*
Imeglimin + Metformin vs. Reference	-0.22	0.08	-2.55	0.012*
Imeglimin Monotherapy vs. Reference	-0.05	0.07	-0.75	0.45
Imeglimin + Other OHA vs. Imeglimin Mono	-0.23	0.08	-2.86	0.006*
Imeglimin + Metformin vs. Imeglimin Mono	-0.17	0.07	-2.15	0.03*

*p < 0.05, **p < 0.001 indicate statistical significance.

*Post hoc* analysis at 6 months showed that Imeglimin + Other OHA (-0.28%, p = 0.004) and Imeglimin + Metformin (-0.22%, p = 0.012) groups had significantly greater HbA1c reductions than the reference group. The Imeglimin monotherapy group did not differ significantly (-0.05%, p = 0.45). Both combination therapies also reduced HbA1c more than monotherapy (Imeglimin + Other OHA: -0.23%, p = 0.006; Imeglimin + Metformin: -0.17%, p = 0.03). These results show superior glycemic improvements with Imeglimin-based combinations over 6 months.

## Discussion

### Structural and mechanistic insights

Imeglimin, developed in Japan, is a novel oral agent for type 2 diabetes that uniquely targets both insulin secretion and mitochondrial health. Imeglimin and metformin both share a common biguanide core structure, characterized by linked guanidine groups. However, Imeglimin is structurally distinguished by the addition of a tetrahydrotriazine ring, forming a cyclic derivative of the biguanide scaffold. This cyclic modification alters its molecular conformation and physicochemical properties, which contribute to its unique pharmacological profile compared to the simpler, linear structure of metformin. Metformin primarily lowers glucose by mildly inhibiting mitochondrial complex I in the liver, reducing hepatic glucose production via AMPK activation. Imeglimin exerts a unique dual action in type 2 diabetes mellitus by simultaneously amplifying glucose-stimulated insulin secretion from pancreatic beta-cells (by improving their mitochondrial function and preserving beta-cell mass) and enhancing insulin action in peripheral tissues (improving insulin sensitivity in the liver and muscle, and reducing hepatic glucose output), thereby addressing both the insulin deficiency and insulin resistance characteristic of the disease (9). Theurey et al. (2022) showed that Imeglimin, unlike metformin, did not induce lactic acidosis in animal models, even under stress or renal impairment. This was attributed to its weaker inhibition of mitochondrial complex I and preservation of mGPDH activity, suggesting a lower risk of lactate accumulation and a safer metabolic profile (16).

This study evaluated the efficacy of imeglimin monotherapy and combination therapies in type 2 diabetes mellitus (T2DM) patients, focusing on glycemic control, mitochondrial function, and metabolic markers over six months. The findings indicate that Imeglimin monotherapy was insufficient for glycemic control, whereas its combination with metformin or other oral hypoglycemic agents (OHAs) significantly improved metabolic outcomes. Previous preclinical studies have shown that Imeglimin improves mitochondrial function through mechanisms such as enhanced oxidative phosphorylation, preservation of mitochondrial membrane potential, and reduction in reactive oxygen species. Aoyagi et al. (2024) demonstrated that Imeglimin improved mitochondrial quality control in pancreatic β-cells of db/db mice by reducing reactive oxygen species and dysfunctional mitochondria through enhanced mitophagy. Unlike metformin, Imeglimin also restored insulin secretion and reduced β-cell apoptosis, supporting its role in preserving β-cell function in type 2 diabetes (12). Sanada et al. (2022) reported that Imeglimin enhanced glucose-stimulated insulin secretion, improved glycemic control, and exerted protective effects on pancreatic β-cells in diabetic mouse models. Chronic treatment improved mitochondrial morphology, increased insulin granule content, and reduced β-cell apoptosis, highlighting Imeglimin’s direct role in preserving β-cell function in type 2 diabetes (17). Kato et al. (2025) demonstrated that Imeglimin protected Schwann cells from mitochondrial dysfunction caused by glucose fluctuations. In IMS32 cells, Imeglimin reduced mitochondrial reactive oxygen species, preserved mitochondrial membrane potential—critical for ATP synthesis and overall mitochondrial integrity—and restored ATP production under both hyperglycemic and hypoglycemic conditions. These results highlight Imeglimin’s potential in mitigating mitochondrial-driven cell death in diabetic neuropathy (18). Our clinical findings align with these experimental observations by demonstrating an increase in mitochondrial DNA copy number in patients receiving Imeglimin-based therapy. This may reflect enhanced mitochondrial biogenesis *in vivo*, although the absence of direct functional assays limits mechanistic interpretation. Nonetheless, our results provide early translational evidence supporting the mitochondrial benefits of Imeglimin previously observed in animal and cellular models.

### Glycemic control

**Figure 2:**
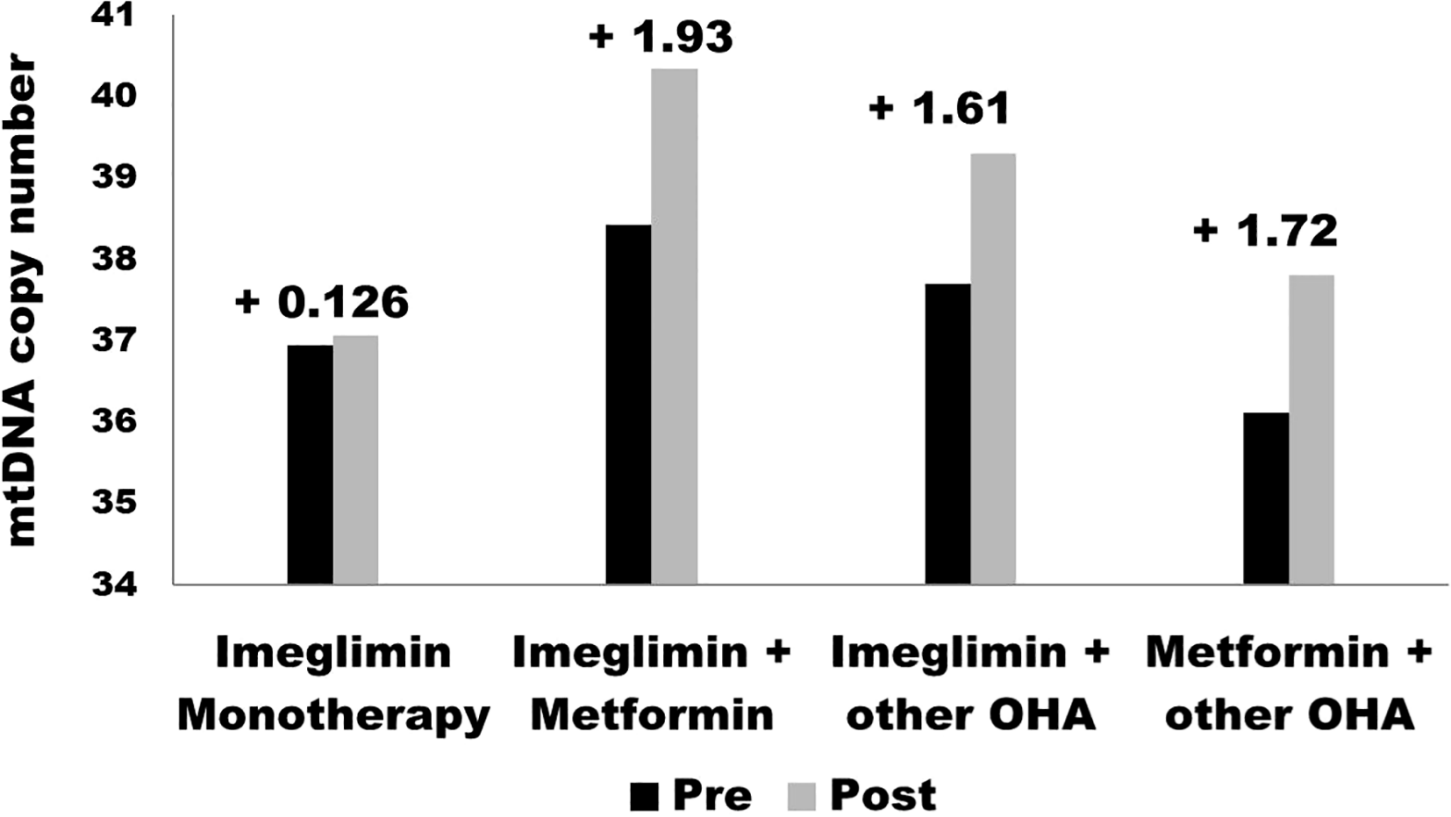
Changes in mtDNA copy number pre- and post-treatment across therapy groups.

HbA1c decreased in the Imeglimin monotherapy group with a weak statistical significance (p = 0.67), suggesting that Imeglimin alone may not sustain glycemic control effectively over time as a monotherapy. These outcomes of the study align with previously reported data from the TIMES 1 and TIMES 2 trials (19, 20). These findings suggest that Imeglimin may have limited efficacy when used as a standalone agent for moderate to severe hyperglycemia and may be more appropriately utilized in combination with other oral anti-diabetic drugs. The reduced efficacy observed in the monotherapy group may be partially attributed to lower baseline HbA1c levels and potential patient heterogeneity, both of which could have influenced the glycemic response. Additionally, the modest effect may reflect reduced residual β-cell function in individuals with longer disease duration, suboptimal adherence to lifestyle measures, and the limited impact of Imeglimin on peripheral insulin resistance, which remains a major contributor to hyperglycemia in T2DM. In contrast, Imeglimin + Metformin and Imeglimin + other OHA significantly reduced HbA1c (p < 0.001), with Imeglimin + other OHA showing the greatest reduction. These results align with prior studies where Imeglimin exhibited enhanced efficacy when combined with metformin due to complementary mechanisms targeting mitochondrial bioenergetics and insulin sensitivity. In contrast to our findings, P. Fouqueray et al. found that co-administration of Imeglimin slightly reduced Metformin exposure and renal elimination but had no clinically relevant impact on Metformin or SITA pharmacokinetics. Systemic exposure to Imeglimin remained consistent with prior studies (21). In support of current findings K. Nishiyama et al. examined the effects of Imeglimin, metformin, or their combination on β-cells, the liver, and adipose tissues in db/db mice. While glucose tolerance and insulin sensitivity remained unchanged, combination therapy restored insulin secretion, increased β-cell mass, and reduced apoptosis, suggesting a protective role in type 2 diabetes treatment (22). A double-blind, placebo-controlled phase 3 trial in Japan assessed the efficacy and safety of Imeglimin (1,000 mg twice daily) in type 2 diabetes patients over 24 weeks. Imeglimin significantly reduced HbA1c by 0.87% compared to placebo (p < 0.0001) with a comparable safety profile, supporting its potential as a treatment option (19). Another 52-week, phase 3 open-label trial assessed Imeglimin’s safety and efficacy as monotherapy or combination therapy in 714 Japanese type 2 diabetes patients. Imeglimin reduced HbA1c by 0.46%-0.92%, with the greatest reduction in combination with DPP4 inhibitors. It was well-tolerated, with no serious drug-related adverse events observed (20). The bar graph in **Figure 2** presents the pre- and post-treatment values for four groups in the study: Imeglimin Monotherapy, Imeglimin + Metformin, Imeglimin + Other Oral Hypoglycemic Agents (OHA), and Metformin + Other OHA. The observed changes indicate that the greatest improvement was seen in the Imeglimin + Metformin group (+1.93), followed by Metformin + Other OHA (+1.72) and Imeglimin + Other OHA (+1.61). Imeglimin Monotherapy showed the least improvement (+0.126). These findings suggest that combination therapies, particularly Imeglimin with Metformin, are more effective in enhancing the measured parameter compared to monotherapy. (**Figure 2**).

Previous studies, including the TIMES 2 trial, have reported gastrointestinal adverse effects with Imeglimin, especially when combined with metformin (20, 23). Our study did not record any notable adverse events during the six-month follow-up, including among patients receiving Imeglimin in combination with metformin. While this is encouraging, the relatively small cohort and limited duration restrict the strength of conclusions that can be drawn regarding overall safety. Previous clinical trials have reported gastrointestinal intolerance with Imeglimin, especially when used in combination regimens, which we did not observe in our population. This discrepancy may reflect demographic or clinical differences in tolerability, or limitations in the sample size to detect rare or delayed adverse effects. As such, broader and longer-term studies are essential to confirm the safety profile of Imeglimin in diverse patient populations.

### Mitochondrial function

**Figure 3:**
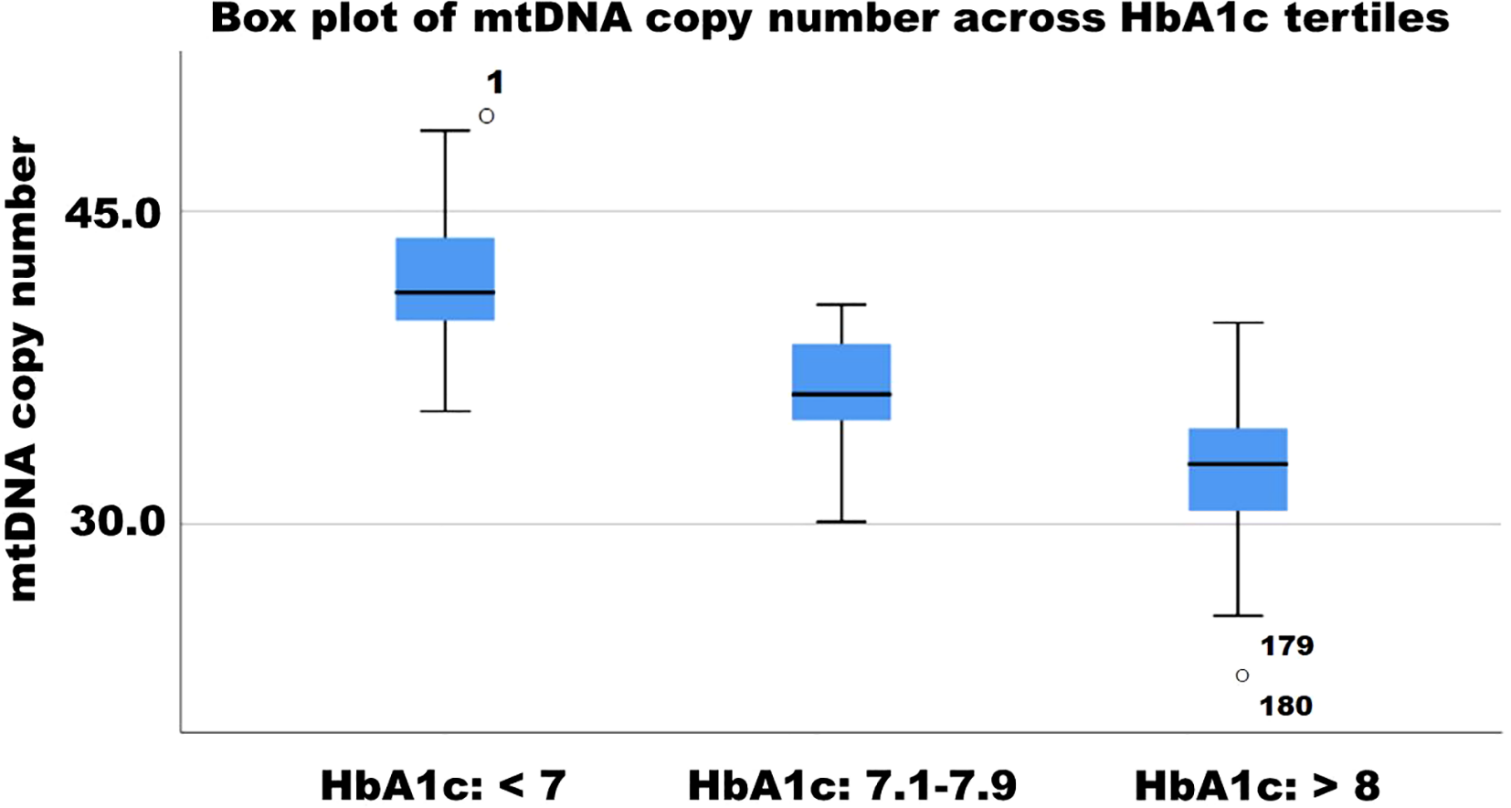
Box plot of mtDNA copy number across HbA1c tertiles.

mtDNA copy number significantly increased in the Imeglimin Monotherapy group (Estimate: -0.4, p < 0.112), whereas Imeglimin + Metformin and Imeglimin + other OHA showed significant increases (both p < 0.001, respectively). These findings suggest that combination therapies, particularly those including metformin, may support mitochondrial biogenesis and function. The negative correlation between mtDNA copy number and HbA1c reduction (r = -0.30, p = 0.006) further reinforces the link between mitochondrial health and glycemic control. Earlier studies have suggested a decrease in mitochondrial DNA copy number in the pathology of type 2 diabetes and its complications, indicating mitochondrial dysfunction as a contributing factor (24–26). (27) However, no preclinical or clinical study has yet assessed changes in mitochondrial DNA copy number in patients with type 2 diabetes undergoing Imeglimin therapy. The box plot illustrates the distribution of mitochondrial DNA copy number (mtDNAcn) across HbA1c tertiles (<7, 7.1–7.9, and >8). A declining trend in mtDNAcn is observed with increasing HbA1c levels, suggesting an inverse relationship between glycemic control and mitochondrial content. The lowest mtDNAcn values are found in individuals with HbA1c >8, with some outliers present. These findings indicate that poorer glycemic control may be associated with mitochondrial dysfunction, reinforcing the role of mitochondrial health in diabetes progression (**Figure 3**). Although Imeglimin has been shown in preclinical studies to exert direct effects on mitochondrial function and enhance β-cell survival, our findings suggest that the observed increase in mtDNA copy number is more plausibly a secondary effect driven by improved glycemic control rather than a direct mitochondrial action. This parallel trend implies that the improvement in mitochondrial biogenesis may be attributed to the metabolic benefits of better glycemic control. Mitochondrial DNA copy number serves as a surrogate marker of mitochondrial content and biogenesis, but does not directly reflect mitochondrial functional capacity. Without accompanying assessments such as ATP production, respiratory capacity, or oxidative stress markers, the link between Imeglimin and mitochondrial function remains speculative (28). In the absence of such measurements, it is not possible to distinguish whether the mitochondrial changes are a direct effect of Imeglimin or an adaptive response to improved systemic metabolic status. To more accurately assess mitochondrial function in clinical settings, future studies could incorporate novel and validated assays such as high-resolution respirometry, to measure mitochondrial respiratory capacity, extracellular flux analysis for real-time assessment of ATP production and oxygen consumption rate, and quantification of circulating mitochondrial-derived reactive oxygen species using electron spin resonance or fluorescence-based assays (29–31).

### Lipid and renal markers

Changes in lipid parameters and renal function markers, including LDL-C, triglycerides, and UACR, were not statistically significant across groups. However, relative telomere length (RTL) was negatively correlated with LDL-C (r = -0.24, p = 0.027), suggesting a potential link between lipid metabolism and cellular aging. Given the structural and mechanistic similarity between Metformin and Imeglimin, assessing relative telomere length (RTL) in Imeglimin-treated T2DM patients is justified. Metformin has been shown to attenuate telomere shortening via AMPK/TERT pathways and reduce cellular senescence. Investigating RTL may reveal Imeglimin’s potential anti-aging effects in T2DM (32–34). Although Imeglimin significantly improved glycemic control when used in combination therapy, no significant changes in relative telomere length (RTL) were observed in any treatment group over six months. This suggests that Imeglimin may not exert short-term effects on telomere dynamics, which typically reflect long-term cellular stress and aging. The weak inverse correlations of RTL change with LDL-C (r = –0.24, p = 0.027) and age (r = –0.22, p = 0.039) support the idea that telomere biology is more closely linked to chronic metabolic stress than to short-term glycemic changes. The lack of measurable changes in relative telomere length over the six-month follow-up period may reflect the inherently gradual nature of telomere dynamics, which typically require extended durations to detect meaningful alterations. In this study, telomere length was included as an exploratory endpoint to preliminarily assess the potential anti-aging effects of Imeglimin, given its known role in reducing oxidative stress and improving mitochondrial function—both key drivers of telomere attrition (35). To strengthen future investigations, longer-term studies incorporating additional molecular markers—such as telomerase activity, DNA damage at telomeric regions, epigenetic modifications, and indicators of chronic low-grade inflammation (inflammaging)—are recommended to more comprehensively assess the impact of diabetes therapies on biological aging processes. A. Baragetti et. *al.*investigated the relationship between LDL cholesterol and leukocyte telomere length (LTL) in genetically confirmed familial hypercholesterolemia (HeFH), clinically diagnosed but genetically unconfirmed FH (CD-FH), and normocholesterolemic controls. HeFH subjects had shorter LTL, particularly in younger and statin-naïve individuals. Additionally, HeFH showed lower circulating hematopoietic precursors, suggesting early cellular senescence (36). The causal relationship between lipids, apolipoproteins, and telomere length (TL) remains unclear. Another study used two-sample Mendelian randomization (MR) with univariate and multivariate approaches. Univariate MR suggested a positive association between certain lipids and TL, but multivariate MR did not confirm this, indicating preliminary evidence (37).

### Clinical implications

The results highlight the limited efficacy of Imeglimin monotherapy and emphasize the need for combination therapy in optimizing glycemic and mitochondrial outcomes in T2DM. Imeglimin demonstrated a favorable safety profile, with no serious adverse events reported. The only noted adverse effect was mild shoulder pain in two participants (1.1%), which was self-limiting. This aligns with previous studies, which have also reported Imeglimin as well-tolerated in T2DM patients. These findings further support its clinical safety in real-world settings. The findings also suggest that patients with lower baseline HbA1c (<7.5%) had greater increases in mtDNA copy number, indicating that early metabolic control may enhance mitochondrial adaptations.

### Limitations and future directions

The study is limited by its short duration (6 months) and lack of direct mitochondrial functional assays. Future studies should explore long-term effects, mechanistic pathways, and patient stratification based on baseline metabolic profiles to optimize therapeutic strategies. The Imeglimin + other OHA group included diverse drugs with differing effects on mitochondrial function. Previous studies suggest that sodium-glucose co-transporter-2 inhibitors directly improve mitochondrial biogenesis, reduce oxidative stress, and enhance ATP production (38). In contrast, there is no clear evidence of a mitochondrial effect for sulfonylureas. Dipeptidyl peptidase-4 inhibitors, while lacking clinical validation, have shown promising preclinical evidence suggesting potential mitochondrial benefits. This heterogeneity may confound interpretation of mitochondrial outcomes and supports the need for stratified analyses or more uniform treatment arms in future studies (39). While mtDNA copy number is an established biomarker of mitochondrial content, it does not directly reflect mitochondrial function. The absence of functional assays such as measures of ATP production or oxidative phosphorylation represents a limitation of our study. Future investigations incorporating these endpoints are warranted to elucidate the full impact of Imeglimin-based therapies on mitochondrial bioenergetics. In our study, relative telomere length did not change significantly over six months, which aligns with the understanding that telomere attrition is a slow process unlikely to be captured over short intervals. While the findings are not conclusive, the telomere analysis was included to explore broader aging-related mechanisms in T2DM. To better evaluate telomere length changes in this study, assessment of telomerase activity and additional markers of telomere maintenance—such as shelterin complex proteins or DNA damage at telomeres—should have been included to provide more mechanistic insight and enhance interpretability.

## Conclusion

Combination therapy with Imeglimin + Metformin or other OHAs provides superior glycemic control and mitochondrial benefits compared to monotherapy. These findings reinforce the role of mitochondrial health in diabetes management and suggest that Imeglimin should be used alongside other agents for maximal clinical benefit.

## Data Availability

The original contributions presented in the study are included in the article. Further inquiries can be directed to the corresponding author(s).
